# Advantages and Challenges of Differential Immune Cell Count Determination in Blood and Milk for Monitoring the Health and Well-Being of Dairy Cows

**DOI:** 10.3390/vetsci9060255

**Published:** 2022-05-27

**Authors:** Sabine Farschtschi, Martin Mattes, Michael W. Pfaffl

**Affiliations:** Division of Animal Physiology and Immunology, TUM School of Life Sciences Weihenstephan, Technical University of Munich, 85354 Freising, Germany; martin.mattes90@web.de (M.M.); michael.pfaffl@tum.de (M.W.P.)

**Keywords:** differential cell count, differential leukocyte count, somatic cell count, dairy cow, immunomonitoring, immunophenotyping

## Abstract

A key challenge of the 21st century will be to provide the growing world population with a sustainable and secure supply of food. Consequently, the dairy farming’s primary task is to lower milk losses and other inefficiencies associated with diseased cows. Moreover, a shift from curative to preventive health management would be desirable for mastitis and a wide variety of other infectious and non-infectious cattle diseases, some of which are known to have profound negative effects on the performance and well-being of cows. Differential cell counting (DCC), a procedure that aims to determine the proportions of different somatic cell types in raw milk samples, has not only the potential to optimize mastitis diagnostics, but it could furthermore serve as a diagnostic tool for monitoring the general and overall health status of dairy cows. Based on a broad search of the literature, the practical utility of various types of DCC is summarized and discussed in this review. Since it might be of advantage to interpret DCC with the aid of data from studies in humans, differences between the immune systems of humans and dairy cattle, with a special focus on surface marker expression profiles and γδ (gamma delta) T-cell characteristics, are also described.

## 1. Introduction

The somatic cell count (SCC) is defined as the sum of all body cells that are found in raw milk. In healthy dairy cattle, this eukaryotic milk cell population is usually composed of more than 97% leukocytes and less than 3% mammary epithelial cells (MECs) [1,2]. Leukocytes can be found in the milk of healthy udders because they cross the blood–milk-barrier (diapedesis) as part of the natural immune surveillance to protect the mammary gland from infections. MECs are shed into the milk as a result of the milk secretion process and due to the continuous renewal of the mammary gland epithelium. In contrast to the cell-preserving merocrine milk secretion in the bovine udder, the cell-destructing nature of apocrine milk secretion in the female breast results in a predominance of MECs in human milk [3].

Mastitis, the inflammation of the bovine mammary gland, is characterized by a recruitment of leukocytes—mainly polymorphonuclear leukocyte (PMN)—into the lumen of the udder [2,4,5,6]. This leukocyte migration causes the SCC to rise. For this simple reason, the SCC is internationally primarily used as the fundamental parameter for managing the udder health in dairy herds. It can be estimated on-site and semi-quantitative by eye, e.g., using a California Mastitis Test (CMT), or counted accurately by a cytometer [7].

About 40 member countries of the International Committee for Animal Recording (ICAR) participate in a global cow milk recording system [8]. In addition to the obligatory analysis of milk yields, cow-milk recording includes the quantification of chemical milk components (e.g., protein, fat, lactose, and urea), as well as somatic cells. In the ICAR member country Germany, the SCC of around 3.45 million individual cows are controlled monthly on a voluntary basis as part of the Dairy Herd Improvement (DHI) [9]. Apart from its great value in monitoring udder health, the SCC is regarded as an indicator of raw milk quality. Hence, checking the SCC in dairy cattle is not just a matter of animal welfare and well-being, but there is also a big economic importance to it. In the European Union, for example, a trigger value of 400,000 somatic cells per milliliter milk is set out in regulation (EC) No. 853/2004. Moreover, the SCC is widely used as a health trait within the genetic evaluation of dairy cattle, meaning that a breeding value is predicted and included in the calculation of total merit indices [10].

Beyond the previous achievements, a more elaborated and detailed examination of these multifaceted cells is promising. In this review, we introduce differential cell counting, a procedure that aims to determine the proportions of different somatic cell types and subpopulations in raw milk samples. Without a doubt, a better understanding of the bovine cellular immune defense (number, function, and tissue distribution of leukocyte subsets) will be crucial for the validation of new DCC-based diagnostic tools. For this reason and knowing that findings in bovine immunology have already been successfully interpreted with the aid of data from studies in humans, we discuss differences between the immune systems of man and dairy cattle, with a special focus on surface marker expression profiles and γδ (gamma delta) T-cell characteristics.

## 2. Differential Cell Count as a Tool to Monitor Mammary Gland Immune Responses

### 2.1. Literature Research and Current Applications

As set out above, the enumeration of somatic cells in bovine milk is an important tool in dairy farming. So, why is there an effort to include the DCC in the evaluation of udder health? This motivation is most likely associated with the results of numerous studies published during the last four decades, in which the DCC was assessed as a more accurate indicator of udder health than the SCC. Some studies, e.g., References [5,6,11], revealed that the option to distinguish between different somatic cell types in raw milk samples could provide additional information for a better diagnosis and treatment of mastitis. Hence, over the years, the scientific community has presented various DCC-based quotients and biomarkers (Table 1) that can be useful in different stages of mastitis. Some can help to detect an early subclinical mastitis, even below an SCC value of 100,000 cells per mL milk [11,12,13]. Pilla et al. [11] calculated the logarithmic ratio of polymorphonuclear neutrophilic leukocytes to lymphocytes. The authors used a cutoff value of 0.495 to distinguish between healthy and diseased quarters. In a different approach, the quantitative relationship between T and B cells was established as a useful parameter for udder health [12]. Soltys et al. [13] proposed the upregulation of CD18 on leukocytes as a sensitive indicator of an early intramammary inflammation. These biomarkers could increase the diagnostic value and treatment efficiency and end the unseen spreading of pathogens from cow to cow, thereby lowering the transmission risk. In addition, this could improve the reproductive performance, as subclinical mastitis is associated with a reduction in the probability of pregnancy [14,15]. Other biomarkers, as, for example, the percentage ratio of granulocytes per macrophages established by Rivas et al. [5], evaluate the stage of infection. This could allow for monitoring the progression of a disease or the success of a treatment. Another important approach to use the DCC is for the distinction between “acute mastitis” and “chronic mastitis” through the determination of highly granulated cells, non-vital cells, and macrophages [16]. This can contribute to the reduction of unnecessary antibiotic treatment, as it is inadvisable for cows with bad chances of bacteriological cure [17]. In order to minimize the development of drug-resistant bacteria, the use of antibiotics in livestock farming should be as low as possible, especially of those that the World Health Organization (WHO) has classified as highest-priority critically important antimicrobials [18]. Furthermore, the DCC can be applied to evaluate a cow’s mastitis susceptibility, for example, by calculating the ratio of CD4+ to CD8+ T cells [19] or by the determination of viability of PMN [20]. Since this susceptibility is partly genetically determined, this could facilitate the election of mastitis resistant cows for breeding programs. To summarize, applying DCC could increase overall animal health and support well-being in dairy cattle.

Besides those approaches, the determination of a DCC of a raw milk sample gained even more attention when automated cell counters were invented for this purpose. In 2013, the first automated on-farm analyzer QScout Farm Lab (Advanced Animal Diagnostics Inc., Morrisville, NC, USA) was launched, which is able to record a three-part DCC [21]. Based on fluorescence microscopy, this machine determines the proportions of neutrophils, lymphocytes, and macrophages in bovine raw milk samples treated with fluorescent dyes. In addition, this instrument offers a mastitis diagnosis. In a study with cows in early and late lactation, Godden et al. [22] compared the obtained automated milk leukocyte differential to common CMT to detect intramammary infections (IMIs) on quarter and cow level and observed only minor differences between the estimates of both methods. The authors also verified the machine’s repeatability. Lozada-Soto et al. [23] also evaluated the usefulness of QScout to enhance the monitoring of udder health in practice. Their results showed a high specificity and a moderate sensitivity. Another automated cytometer, the Fossomatic DC (FOSS Analytical A/S, Hillerød, Denmark), uses flow cytometry [24] to measure macrophages and the combined proportion of PMNs and lymphocytes [25] in up to 600 milk samples per hour. Combined with SCC determination, this method was used to detect intramammary infections in several recent studies [26,27].

### 2.2. Multicolor Flow Cytometric Immunophenotyping

**Figure 1:**
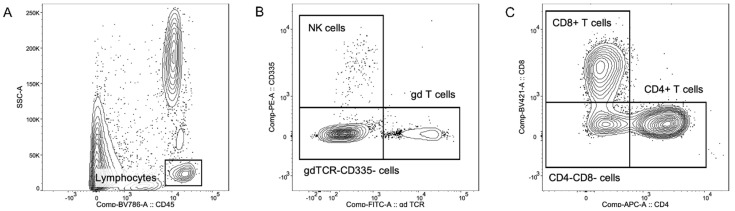
Exemplary gating strategy of lymphocyte subpopulations in milk. Figure and method adapted from Farschtschi et al. [33]. (**A**) Pan leukocyte marker CD45 vs. SSC-A, (**B**) gdTCR vs. CD335, and (**C**) CD4 vs. CD8.

Laser-based flow cytometers analyze individual cells passing through a laser beam at a rate of several thousand events per second. A prerequisite is that the examined cells are intact and suspended in a fluid. Detectors for scattered light provide information on cell size (measured by forward scatter) and cell granularity (measured by side scatter). In addition, fluorescence detectors identify specific structures on (or within) the target cells in the case that these markers have been stained with fluorochromes prior to a flow cytometry measurement. Often used markers for the characterization of immune cells are surface proteins of the CD (cluster of differentiation) system. Approximately 400 different human CD markers have been identified by now [29], some of which are conserved in other species, including non-human mammals (e.g., cattle and mice), fish, and birds [30]. When CD markers or other antigens are stained with a panel of differently fluorescence-labeled antibodies, the whole assay is referred to as multicolor flow cytometric immunophenotyping. In this way, all free circulating cells in blood or milk can be differentiated, meaning that they are individually counted and, hence, could be assigned to different subpopulations with specific characteristics. Test results that reveal altered individual cell counts or the presence of cell populations with unusual phenotypes can be used for diagnostic purposes, if reference ranges have been established. There are recent technological advantages that allow us to measure up to ten or even more fluorochromes in a single run [31,32,33]. Furthermore, over the last years, various commercially available antibodies have been identified or produced that (cross-)react with bovine marker proteins [34]. Thus, researchers are now able to identify the variety of cell subsets in bovine blood and raw milk. An exemplary gating strategy to detect different lymphocyte subpopulations can be seen in Figure 1. In other words, recording extended DCC by multicolor flow cytometric immunophenotyping has become a promising tool for monitoring the general health status and well-being of dairy cows, albeit labor-intensive and expensive.

Certainly, laser-based flow cytometry methods do not always require high-quality antibody conjugates. Membrane permeable or impermeable fluorescent dyes such as SYTO 13 or propidium iodide for staining whole organelles (e.g., cell nuclei), instead of specific antigens (e.g., CD surface molecules), are more cost-effective ways to differentiate somatic cells in bovine milk. However, only the three major types of leukocytes (lymphocytes, macrophages, and neutrophils) and their vitality (apoptotic, necrotic, and viable) are currently distinguishable with these methods [16,35,36]. The differentiation of cells by manual cell counting is also restricted. This standard cytology technique makes use of film preparations, either cytospins or smears, to determine DCCs via light microscopy. The staining is often conducted with mixtures composed of cationic and anionic dyes, e.g., azure B plus eosin Y (Romanowsky-type stains). Though manual cell counting has been shown to work with both blood and milk samples [37,38], it is not suitable for detecting marker proteins that characterize leukocyte subpopulations and their differentiation stages (e.g., cytotoxic T cells, intermediate monocytes, or memory B cells). In contrast, flow cytometric immunophenotyping is made for such deeper insights into the biology of immune relevant cells.

## 3. Extended Differential Cell Counts in Milk as a Tool to Monitor the General Health Status of Dairy Cattle

### 3.1. Background and Basic Assumptions

In contrast to the huge set of immunologic biomarkers that can be used in mastitis diagnostics, specific DCC patterns in other infectious or non-infectious cattle diseases have hardly been studied so far. Interestingly, depending on the geographical area, the prevalence of these diseases can exceed the clinical mastitis prevalence by far [39]. Without doubt, efforts to improve mastitis diagnostics are important, since udder infections, notably subclinical mastitides, are still one of the most prevalent and costly diseases in the dairy farming with the largest losses [40,41]. In an average Dutch dairy farm, for example, mastitis causes annual costs of up to 240 Euro per cow [42]. Nevertheless, an improved diagnosis of other cattle diseases that give rise to similar economic losses in some countries is needed [43,44,45]. Moreover, it should be remembered that, not only udder infections but also a wide variety of other cattle diseases, e.g., foot and reproduction disorders, cause severe pain and consequently reduce animal welfare [46,47].

When reading the latest textbooks on the bovine immune system, e.g., Schalm’s veterinary hematology, 6th edition [48], it becomes apparent that the fundamentals of immunology, which have been mainly obtained from studies in mice and humans, are also introduced and accepted in bovine diagnostics and medicine. Schilling’s biological leukocyte curve is for example utilized in bovine medicine as a model for blood leukocyte dynamics in response to tissue damage (neutrophilic struggle phase → monocytic defense phase → lymphocytic healing phase), though it has been originally established in human medicine based on the examination of a young woman with puerperal sepsis [49]. We can therefore start from the assumption that a specific cattle disorder, caused by a certain type of pathogen (bacterium, fungus, and virus), elicits a typical immune response with enhanced production, proliferation, differentiation, or activity of a corresponding specialized leukocyte subset—just as we observe it in mice and humans. For example, some infections with parasites can be identified based on a rise in the number of eosinophils, since this immune-cell type can destroy multicellular parasites, which are too large for phagocytic elimination, via releasing cytotoxic substances (degranulation) [50]. However, it is not clear to what extend this knowledge also holds true for the same cell populations in milk.

### 3.2. Similarities between Differential Cell Counts in Milk and Blood in Healthy Cattle

Published data on the proportions of leukocyte subsets in peripheral blood of healthy cows are largely consistent, as can be seen by means of selected examples shown in Table 2 and Table 3. In contrast, published data on the proportions of somatic cell subpopulations in bovine raw milk differ strongly. There are two major reasons for the difficulty to differentiate somatic cell populations by flow cytometric immunophenotyping or standard cytology. First, the morphology of phagocytes alters due to the incorporation of milk components after diapedesis [51,52]. Second, the viability of all immune cell populations [53] decreases, presumably caused by intrinsic factors (e.g., advanced cell age and depletion of energy reserves in the course of diapedesis) and adverse environmental factors in milk [54].

Standards for DCC in milk need to be determined with respect to the fact that there are plenty of factors that could influence test results. The consequent lack of generally accepted reference intervals for DCC in bovine milk is one of the reasons why this parameter has not yet been included in the DHI. There is not even a scientific consensus about the most abundant bovine cell type in milk of healthy udders. With some exceptions, studies in dairy science revealed a predominance of either macrophages or lymphocytes in milk of mastitis-free cows. Schwarz et al. [6] not only presented a link between extremely low SCC values (≤6250 cells/mL) and very high lymphocyte percentages of up to 88%, but also a predominance of lymphocytes throughout the whole SCC range of healthy udder quarters (SCC ≤ 100,000 cells/mL). In contrast, investigations performed by Sarikaya et al. [67] point to a threshold within the SCC range of healthy udder quarters above which macrophages outnumber lymphocytes. Damm, et al. [25], who used a different method to prepare the samples for the subsequent flow cytometric analysis, described the number of lymphocytes to remain rather constant and the proportion of macrophages to decrease as SCCs increased. A temporary predominance of neutrophils in bovine raw milk during the course of an udder infection, marked in the early inflammatory phase [5] and observable in clinical cases [4], as well as some subclinical cases [6], is beyond dispute.

There is a central question to be answered prior to the usage of differential cell counting for diagnostic purposes: Can changes in the differential blood cell count, e.g., elicited by a specific organ disease, also be seen in the milk DCC?

To our knowledge, there is virtually no data available on milk DCC patterns in non-infectious cattle disorders such as metabolic diseases (e.g., ketosis and ruminal acidosis) or specific organ diseases (e.g., abomasal ulcers and fatty liver), though the respective changes of the blood leukogram have partly been identified, as reviewed by Roland et al. [68]. However, at least for some systemic infections with, for example, MAP (*Mycobacterium avium* subspecies *paratuberculosis*) or BLV (bovine leukemia virus), quantitative changes of leukocyte subsets have been reported in both bovine blood and milk samples [69,70]. Farschtschi et al. [33] used three different vaccines (against bovine respiratory disease, trichophytosis, and bovine viral diarrhea) as immune stimuli to examine the effect of systemic immune reactions with a high-resolution DCC (HRDCC) that detects ten subpopulations of immune cells in addition to the main populations, as well as their viability. The authors could show the impact on both milk and blood HRDCCSs. In their statistical analysis, CD8+ T cells, B cells, and monocyte/macrophage subpopulations were among the most influential factors.

In addition to their ability to recognize PAMPS (pathogen-associated molecular patterns), innate immune cells also respond to DAMPS (damage-associated molecular patterns) that are released from necrotic cells [71]. Therefore, inflammatory reactions can be elicited by biological agents (e.g., microbes), chemical agents (e.g., acids), and mechanical agents (e.g., pressure), as long as tissue damage occurs. With this in mind, one can assume that, not only infections, but also some cytopathic processes in non-infectious cattle disorders modify the quantity or the phenotype of leukocyte subsets in bovine body fluids. Most likely, a small proportion of these modifications are usable as highly sensitive and specific diagnostic, prognostic, or predictive biomarkers. Inflammatory neutrophilia or lymphopenia are examples of poor biomarkers, since they are seen in many cattle disorders [68]. A promising biomarker would be a change in the quantity or marker expression profile of a particular leukocyte subpopulation, e.g., a lymphocyte subset, which is unambiguously linked to a specific pathological event. Nonetheless, one should always consider that leukogram changes do not even have to be related to tissue damage. Apart from the enhanced production (bone marrow), proliferation, and extravasation of leukocytes in cows with inflamed tissues, other mechanisms can trigger temporary variations of leukocyte numbers in healthy animals. For example, there is a link between hypertension during stress situations and a shift of blood leukocytes from the marginal to the circulatory pool. This phenomenon is known as physiologic leukocytosis [68].

## 4. Differences between the Immune Systems of Humans and Dairy Cattle

### 4.1. Background and Basic Assumptions

The list of differences between the human and murine immune system presented in review papers is long [72,73,74]. In contrast, our knowledge on differences between the immune systems of ruminants and humans (or mice) is relatively poor, as reviewed by Bailey et al. [75] and Entrican et al. [76]. The primary reason for this can be found in the low—albeit currently growing—number of commercially available antibodies against ruminant proteins, as this limits immunologic studies in these animals. What we know from phylogenetic analyses is, however, that rodents, primates, and ruminants diverged around 90 million years ago [77]. During this incredibly long period of time, the immune systems of mice, humans, and cattle must have undergone multiple modifications in order to adapt to specific environments inhabited by distinct types of steadily changing pathogens. Visible signs of adaptation are, for example, marked differences in body size (small vs. mid-size vs. big) and diet (omnivore vs. ruminant). Further evidence for unique surviving strategies can be found in the genome sequences of the mammal species. Compared to mice and humans, cattle possess an elevated copy number of certain immune-related genes including interferons, defensins, and T-cell receptor (TCR) V segments [78]. Gene duplications such as these are known to generate functional novelty, e.g., by providing new genetic material for mutations or higher gene product dosages [79]. Therefore, they might enable cows to build up strong and diverse immune responses that cope with the high number of ruminal microorganisms, some of which are opportunistic pathogens, e.g., *Fusobacterium necrophorum* [80].

The concentration of peripheral blood leukocytes is quite similar in humans and cattle. For cattle, a total leukocyte count (TLC) within 5000 to 13,000 leukocytes per µL is regarded as physiological [68]. The human TLC reference interval ranges from about 4000 to 11,000 leukocytes per µL [81]. Human blood usually contains smaller amounts of eosinophils and basophils than bovine blood. However, the blood monocyte count of humans is often a bit higher and less variable [55]. The main difference that can be extracted from a five-part differential blood cell count of human and cattle, standardly measured by automated hematology analyzers that use, for example, the “coulter principle” [82] in combination with laser-based flow cytometry techniques, is the neutrophil-to-lymphocyte ratio. Typically, cattle have more than 50% lymphocytes within their blood leukocyte population, whereas more than half of the circulating leukocytes in healthy humans are neutrophils [55].

Without reference data, it is difficult to interpret laboratory test results correctly. In human medicine, a huge body of empirical data is available for the interpretation of (abnormal) differential blood counts determined by multicolor flow cytometry [37,83,84]. Thanks to this high-resolution method, our knowledge on the function and complexity of leukocyte subsets in human breast milk is also steadily growing [85,86]. In bovine medicine, however, the actual role of several leukocyte subsets is still unknown—even their function in blood. This is not surprising, given that, for example, subpopulations of peripheral blood monocytes (classical and non-classical monocytes) have been known in men since 1989 [87], whereas these cells have been described in cattle for only a couple of years ago [88]. Moreover, human natural killer (NK) cells were discovered in the early 1970s (reviewed by Boysen et al. [65], for example), whereas a precise characterization of bovine NK cells was only presented in 2004 [89]. So, why should we not use the huge amount of empirical data from human medicine for the interpretation of the DCC in blood or milk in cattle? Clearly, this would require a deeper understanding of similarities and differences between the human and bovine immune system. Otherwise, diagnoses would be inaccurate or false. Progress in comparative immunology could also facilitate the development of new pharmaceuticals in bovine medicine such as vaccines and antimicrobials. There is also a chance to uncover the potential of cows as animal models in human medicine. Advantages of sheep models over mouse models in this field have already been reviewed [76].

### 4.2. Humoral Responses, Architecture, and Surface Marker Expression Profiles

Unique features within the humoral arm of the bovine innate and adaptive immune system, for example, production of novel chemokines [90], preferential use of lambda light chains in antibodies [91], IgG1 secretion into milk, and lack of transplacental transfer of antibodies into the fetus [92], have already been revealed. This extends to the architecture of the bovine immune system, which is, for example, characterized by a small bone-marrow storage pool for granulocytes [68] and an expanded gut-associated lymphoid tissue (GALT) with a continuous ileal Peyer’s patch [93,94]. The polarization of bovine T helper cells to T_H_1 or T_H_2 is generally weak [95] but does exist in some diseases, such as mycobacterial infections [96,97]. In this regard, cattle act more similar to humans (moderate T-cell polarization) than mice (strong T-cell polarization) [73]. Nevertheless, especially our growing knowledge on bovine γδ T cells, which are discussed later in this review, points to a quite unique cellular defense in cattle concerning not only tissue distribution but also number and function of certain leukocyte subsets. The differences in the expression of nine surface markers on human and bovine leukocytes are presented in Table 4 and Table 5.

Since leukocyte surface markers act as receptors or ligands for cell communication and cell-substrate adhesion, they define the functionality of a given cell. With few exceptions, these markers are expressed by various cell subsets. For example, bovine CD8 is not only expressed on cytotoxic T cells, but also on subsets of γδ T cells and NK cells [102,103,121]. Thus, when recording extended DCC in bovine body fluids by means of flow cytometric immunophenotyping, only the examination of certain marker combinations in a single tube will provide accurate data on the identity, quantity, differentiation status, activation state, and maturation stage of target cell populations.

## 5. The Special Role of γδ T Cells in Bovine Immune Responses

There are two major differences between human and bovine γδ T cells which highlight the importance of these lymphocytes in cattle: Firstly, γδ T cells constitute 15% (cows) to 60% (newborn calves) of all circulating lymphocytes in cattle, whereas it is unusual to find more than 5% gamma delta T-cell receptor (gdTCR+) cells within the population of human peripheral blood lymphocytes [31]. Similar to their murine counterparts, human γδ T lymphocytes have a propensity for epithelial surfaces, where they act as sentinel cells [122]. Secondly, the gdTCR repertoire is much bigger in cattle than in humans, which may enable bovine γδ T cells to bind a greater variety of antigens. For example, the number of V gene segments that encode a part of the variable region of TCR δ chains (referred to as Vδ segments) is about 19 times higher in cattle compared to in humans [123]. This quantitative difference becomes important due to the fact that bovine γδ T cells also show a highly diverse usage of their Vδ segments in many tissues [124]. In contrast, human γδ T cells show a tissue-specific usage of Vδ segments, which lead to their subdivision into a Vδ1+ cell population, present at mucosal surfaces, and a Vδ2+ cell population, located in the peripheral blood [106,125].

In cattle, three γδ T cell subsets have been described based on differences in the expression of a particular surface marker. In contrast to how it is in humans, this marker is not a TCR δ chain but a transmembrane glycoprotein that belongs to the scavenger receptor cysteine-rich superfamily (SRCR), the Workshop Cluster 1 (WC1) molecule. Wang et al. [108] suggested a role of bovine WC1 as a co-stimulatory molecule for the TCR, similar to that of CD4 and CD8 in the αβ T-cell population. This is interesting, since WC1 is known to be functionally expressed in other ruminants but not in humans [126].

Bovine γδ T cells that express WC1 on their surface (WC1+ cells) are located in peripheral blood, skin, and peripheral lymph nodes, whereas WC1− cells can be found in other tissues, such as the spleen, gut, and mammary gland [64,103,127]. Within the WC1+ cell population, a further differentiation into WC1.1+ and WC1.2+ cells is possible based on the expressed isoform of the WC1 molecule [128,129]. When bovine CD4+CD25highFoxp3+ cells proved to be non-regulatory in ex vivo experiments, it was suggested that γδ T-cell subsets might fill in the role of Tregs in cows [130]. Guzman et al. [31] supported this view by showing that WC1− cells produce the anti-inflammatory cytokine interleukin 10 (IL-10) and suppress αβ T-cell proliferation ex vivo. Moreover, Hedges, et al. [131] interpreted the gene-expression profile of WC1− cells as regulatory. Their transcriptome analysis furthermore revealed a pro-inflammatory role of WC1+ cells. However, according to more recent ex vivo experiments, only WC1.1+ cells produce high amounts of the pro-inflammatory cytokine interferon gamma (IFN-γ) upon stimulation, whereas activated WC1.2+ cells display a regulatory phenotype similar to that of WC1− cells, with suppression of αβ T cells via IL-10 production [31].

Within the Vδ2+ cell population in human peripheral blood, IFN-γ producing γδ T lymphocytes [132] and those that act as regulatory cells [133] can be found. However, as reviewed by Pang et al. [106], the functional plasticity of activated Vδ2+ cells is far more diverse and dependent on the cytokine milieu, in which the antigen contact occurs. Summarizing the body of knowledge on these immune cells from ex vivo studies, Vδ2+ cells cannot just act TH1-like or Treg-like but also TH2-like (production of IL-4) [132], TH17-like (production of IL-17) [134], TFH-like (stimulation of follicular B cells) [135], and TAPC-like (expression of MHC-II for antigen presentation) [136]. To a lesser extent, human Vδ1+ cells also show diverse functions, such as tumor-directed NK-like cytotoxicity [137] or promotion of tissue repair [138].

Bovine γδ T cells are quite similar to human γδ T cells when it comes to functional heterogeneity. Similar to their human counterparts, they are able to act both as innate and adaptive immune cells. Equipped with these multiple functions, human and bovine γδ T cells can fight against a wide range of pathogens, as reviewed by Holderness et al. [139]. Infections in cattle with known involvement of γδ T cells are, for example, *Salmonella enterocolitis* [140], foot-and-mouth disease [104], enzootic bovine leucosis [141], and paratuberculosis [70]. Paratuberculosis or Johne’s disease is a chronic enteritis of ruminants, caused by MAP. The disease occurs worldwide and causes considerable economic losses in dairy cattle due to reduced milk production and premature culling [142]. When Badi et al. [70] investigated the role of different lymphocyte subsets in subclinical MAP infections, they discovered that only the number of γδ T cells was increased in both milk and blood samples of diseased animals. Therefore, the number of γδ T cells could be useful as a marker for the early detection of MAP infections in dairy herds. Moreover, the number of WC1+ cells, which express IL-10, has been shown to increase in cattle with a clinical form of MAP infection compared to subclinical or non-infected ones [143].

The role of γδ T cells in bovine mammary gland infections is still obscure. We know that WC1+ cells in milk do not contribute to mastitis resistance in cattle [19]. Moreover, Riollet et al. [61] demonstrated that, within the lymphocyte population in bovine milk, the proportion of γδ T cells does not change in the course of chronic *Staphylococcus aureus* mastitis, but the expression of WC1 is downregulated. Since most mastitis-causing pathogens are extracellular in nature, antibody-mediated immune responses are thought to be more important for the resolution of udder infections than cell-mediated immune responses [144]. However, experiments performed by Soltys et al. [13] revealed an elevated number of γδ T cells in milk samples taken from cows with acute staphylococcal or streptococcal infections. Faldyna et al. [64] detected a marked rise in the proportion of γδ T lymphocytes in lavages of stimulated cow udders, which was mainly caused by an active recruitment of WC1+ cells from the peripheral blood. Obviously, further research is needed to clarify the role of γδ T cells in bovine mastitis.

The rumen of cattle contains high quantities of bacteria, so that immune cells, especially those in the GALT, are regularly exposed to great amounts of bacterial structures. Against this backdrop and knowing that PAMPs are not only present in pathogenic microbes but also in the resident microbiota [145], it is comprehensible that a very tight regulation of innate immune responses was favored in the evolution of cows. Candidates for immune suppression and controlled release of pro-inflammatory cytokines only upon recognizing structures of dangerous microbes could be found within the bovine γδ T-cell population. As mentioned above, this highly abundant multifunctional immune cell subset plays a major role in the downregulation of bovine immune responses, e.g., by producing anti-inflammatory IL-10. In addition, γδ T cells are major providers of pro-inflammatory IFN-γ upon activation. Moreover, this activation is unusual: not strictly PAMP-dependent, but including other pathways, such as the recognition of microbial lipid antigens and low-molecular-weight phosphoantigens via the TCR. Interestingly, γδ T cells are stimulated by non-peptide phosphoantigens, even in the absence of APCs [146]. Hence, in contrast to MHC-restricted αβ T cells, γδ T cells can respond immediately to these “danger molecules” that are not only produced by pathogenic bacteria and protozoa [147,148] but which also accumulate in stressed body cells [149]. Releasing a high number of γδ T cells into the circulation might therefore be a key strategy by which cattle and other ruminants handle the high burden of microorganisms in their digestive tract.

In addition to their roles in the regulation of immune responses or the direct killing of tumors and invaders, some bovine γδ T cells may have tissue-repair capacity. This assumption is based on the expression of epidermal growth factor (EGF) mRNA in these cells [150].

## 6. Conclusions

Numerous diseases, undetected stress, and inadequate feeding are major causes for reduced profits in the dairy farming [151,152]. Early monitoring systems that provide information on the general health and well-being status of cows are, thus, desirable. In contrast to mastitis control, the routine diagnosis of other cattle diseases is largely based on physical examinations, meaning that laboratory analyses (hematology, biochemistry, and microbiology) are only performed when needed. In these cases, blood tests are usually favored over milk tests. However, since the dairy farming has implemented a well-performing system in which milk is taken monthly from individual cows (DHI) [7], new methods for monitoring the health status of cattle should focus on this easily available sample material. Multicolor flow cytometric immunophenotyping has become a promising tool for studying somatic cell populations in milk. The detailed characterization and fast quantification of several leukocyte subsets in a single measurement are key features of this method. Some of these specialized immune cells will show alterations in population size upon encountering “their pathogens”, whereas others may respond to non-infectious pathologic processes (e.g., disturbances in the energy metabolism) with phenotypic modifications. Thus, extended DCCs, such as HRDCC, could provide valuable information on a wide variety of infectious and non-infectious diseases (general health status). Unfortunately, the high costs of labeled antibodies required for multicolor flow cytometry are problematic in view of the fact that millions of samples have to be analyzed within national cow milk recording programs. Therefore, multicolor flow cytometric immunophenotyping should be regarded as a screening tool that assists in finding new DCC-based biomarkers, which could be integrated into programs such as the DHI.

## Figures and Tables

**Table 1:** Selected examples of DCC-based quotients or biomarkers that have been suggested for improving mastitis diagnostics. In the cited papers, differential cell counting was either conducted with standard cytology or laser-based flow cytometry techniques. CD = cluster of differentiation.

Biomarker in Bovine Raw Milk	Limit	Information about Udder Health	Reference
T cells per B cell → CD2/CD21 index	<10	Subclinical mastitis	[12]
Log (neutrophils per lymphocyte) → Log PMN/lymphocyte ratio	>0.495		[11]
CD18 expression level on neutrophils and lymphocytes	“High”		[13]
Granulocytes per macrophage → PMN/M ratio	<2.39	Mastitis in resolution phase	[5]
Percentages of macrophages AND granulated cells AND non-vital cells	>4.5%, >25.5%, >22%	Chronic mastitis	[16]
T helper cells per cytotoxic T cell → CD4/CD8 ratio	<1	Low mastitis resistance	[19]
Viability of neutrophils	“Low”		[20]
CD11b expression level on leukocytes	“High”	Udder inflammation	[28]

**Table 2:** Published data on the proportions of different cell types in peripheral blood and raw milk of healthy cows.

Cell Type	Percentage of all Leukocytes in Bovine Peripheral Blood	Percentage of All Somatic Cells in Bovine Raw Milk
PMNs	20–50 [55] 22 [56] 36 [57]	34 [38] 31–50 [1] 9 [58] 41 [28]
Eosinophils	2–6 [55] 8 [56] 5 [57]	<1 [38] <1 [1]
Basophils	0–2 [55]	<1 [38]
Monocytes	2–6 [55] 10 [56]	
Macrophages		35–79 [59] 46 [38] 35–43 [1] 52 [58] 13 [28]
Lymphocytes	45–65 [55] 55 [56]	10–28 [59] 21 [38] 14–26 [1] 46 [28]
Mammary epithelial cells		1–3 [1] <1 [38]

**Table 3:** Published data on the proportions of different lymphocyte subsets in peripheral blood and raw milk of healthy cows. CD = cluster of differentiation; WC1 = workshop cluster 1.

Lymphocyte Subset	Percentage of All Lymphocytes in Bovine Peripheral Blood	Percentage of All Lymphocytes in Bovine Raw Milk
T cells (αβ and γδ)	62 * [60]	40–50 [59] 84 * [60] 90 [13]
αβ T cells	44 * [60] 50 [56]	64 * [60] 54 [61] 80 [13]
CD8^+^ cells	20 * [60] 13 * [19] 20 [56]	40 * [60] 21 [61] 10 * [19] 50 [13]
CD4^+^ cells	24 * [60] 31 * [19] 30 [56]	24 * [60] 33 [61] 31 * [19] 30 [13]
γδ T cells	18 * [60] 15–30 [62] <60 in calves [31] 7–20 [63] 28 [64]	20 * [60] 9 ^#^ [64] 10 [13]
WC1^+^ cells	13 * [19] 5–15 [63] 26 [64]	5 [61] 22 * [19] 1 ^#^ [64]
WC1^−^ cells	2–5 [63] 2 [64]	8 ^#^ [64]
B cells	38 * [60] 44 * [19] 16 [56]	20–25 [59] 8 * [60] 1 [61] 36 * [19]
NK cells	2–10 [65]	2–4 in buffalo raw milk [66]

* Converted from “% of bovine mononuclear cells” to “% of bovine lymphocytes” (simplifying assumptions: fixed number of monocytes/macrophages in the evaluation period and exclusion of not investigated, minor lymphocyte subsets). # Measured in mammary gland lavages from virgin heifers.

**Table 4:** Differences in the surface marker expression of human and bovine lymphocytes. Apart from the indicated references, References [98,99] were investigated to collect the presented data. CD = cluster of differentiation; TCR = T-cell receptor; MHC = major histocompatibility complex.

CD Marker	Major Functions	Expression on Human Leukocytes	Expression on Bovine Leukocytes
CD4	Co-receptor with MHC class-II-restricted TCRs in antigen recognition.	On T cells that recognize antigens associated with MHC class II molecules (T helper cells and regulatory T cells), monocytes, macrophages.	Only on T cells [100].
CD8	Co-receptor with MHC class I-restricted TCRs in antigen recognition.	On T cells that recognize antigens associated with MHC class I molecules (cytotoxic T cells), subsets of γδ T cells, NK cells and monocytes.	Similar [101,102,103].
CD335	Major cytotoxicity-activating receptor (induces the lysis of virus-infected cells and tumor cells).	On NK cells.	- On NK cells [89], but differences with regard to the co-expression of CD16a (co-expressed on all bovine NK cells but not on human NK cells in lymph nodes) [65]. - Inducible on a subset of γδ T cells in acute viral infections [104].
gdTCR	Antigen receptor, e.g., to antigens presented by antigen presenting cells (APCs) via nonclassical MHC-molecules [105].	Only on γδ T cells [106].	Only on γδ T cells [63,107], but differences with regard to the co-expression of Workshop Cluster 1 (WC1), a possible costimulatory molecule for the gdTCR [108] that is exclusively expressed on ruminant γδ T-cell subsets.
CD21	Complement receptor that binds to the breakdown products of Complement component 3 (C3). Associated with CD19 and CD81 (B cell coreceptor complex).	On mature B cells, follicular dendritic cells.	Only on mature B cells [109].

**Table 5:** Pan-leukocyte marker CD45 and differences in the surface marker expression of human and bovine myeloid immune cells and lymphocytes. Apart from the indicated references, References [98,99] were investigated to collect the presented data. CD = cluster of differentiation.

CD Marker	Major Functions	Expression on Human Leukocytes	Expression on Bovine Leukocytes
CD45	Signaling molecule (protein tyrosine phosphatase) that regulates a variety of cellular processes including cell growth, differentiation. Critical requirement for antigen receptor-mediated activation of T cells and B cells.	On all leukocytes.	Similar [57,110].
CD11b	- Subunit of Mac-1 (CD11b/CD18), a complement receptor that binds to iC3b or IgG complement on opsonized targets and mediates the subsequent ingestion process (→ macrophages and neutrophils). - Important for the transendothelial migration of monocytes and neutrophils (interactions occurs with stimulated endothelial cells). - Many other roles (e.g., in chemotaxis and apoptosis).	- On granulocytes, monocytes, macrophages, NK cells and subsets of T cells and B cells. - On mature neutrophils (band cells, segmented cells) and late immature neutrophils (metamyelocytes) but not on early immature neutrophils (promyelocytes, myelocytes) [111]. - Elevated expression level on a subset of mature neutrophils upon lipopolysaccharide (LPS) activation [112].	- On granulocytes, monocytes, macrophages and lymphocyte subsets [107,113] but obviously not on ruminant NK cells [114]. - On mature neutrophils (band cells, segmented cells) but absent on most early and late immature neutrophils (myeloblasts, promyelocytes, myelocytes, metamyelocytes) [115].
CD14	Receptor for complex of LPS and soluble LBP (lipopolysaccharide-binding protein).	High expression level on monocytes and macrophages, weak expression level on granulocytes.	Only on monocytes and macrophages [6,57], in contrast to other ruminant species (sheep and goats) which also show a high CD14 expression on granulocytes [116].
CD16a	Low affinity Fc receptor for IgG2 and IgG3. Binds to IgG on opsonized antigens and mediates phagocytosis or antibody-dependent cellular cytotoxicity (ADCC) plus cytokine production.	On NK cells (in blood), macrophages, γδ T cells and monocyte subsets (nonclassical monocytes (ncM) and intermediate monocytes (intM), not classical monocytes (cM)).	- On NK cells [101], macrophages and monocyte subsets (ncM and intM) [88], but obviously not on γδ T cells [101]. - Similar frequency and phenotype of human and bovine monocyte subsets (6% CD14+CD16++ ncM, 4% CD14++CD16+ intM, 90% CD14++CD16− cM) which may represent differential developmental stages, but differences with regard to function (intM and ncM are pro-inflammatory in humans, whereas intM and cM are regarded as pro-inflammatory in cattle) [88,117]. - Obscure role of bovine ncM: low phagocytic capacity, low mRNA expression of neutrophil-attracting chemokines and neither LPS induced interleukin 1 beta (IL-1β) release nor generation of reactive oxygen species (ROS) [88]. - Bovine ncM show high expression of CD1b (involved in lipid antigen presentation), induce strong allogeneic T cell responses and may also be pro-inflammatory [118].
CD16b	Low affinity Fc receptor for IgG1 and IgG3, similar to CD16a.	On neutrophils, absent in eosinophils (but inducible by interferon gamma, IFN-γ). On all mature neutrophils (band cells and segmented cells), with no elevated expression level upon LPS stimulation [112]. Not on early and late immature neutrophils (promyelocytes, myelocytes, metamyelocytes) and thus a possible marker for early inflammatory responses, especially when CD11b expression is also taken into account [111].	With regard to CD16b expression, bovine neutrophils are similar to equine neutrophils [119] but dissimilar to the largely (>90%) CD16b+ neutrophils in pigs [120], goats, and sheep [116].

## Data Availability

Not applicable.
